# Multimodal Therapeutic Strategies for the Management of Sarcopenia and Frailty in Chronic Obstructive Pulmonary Disease: A Narrative Review

**DOI:** 10.3390/nu18030543

**Published:** 2026-02-06

**Authors:** Saoussen Naas, Monika Fekete, Gabriella Szendro, Tamas Komaromi, Zsolt Rozgonyi, Erik Palmer, Lorinc Polivka, Regina Bakos, Borbala Szalai, Veronika Muller, Janos Tamas Varga

**Affiliations:** 1 Department of Pulmonology, Semmelweis University, 1083 Budapest, Hungary; naassaoussen@gmail.com (S.N.); komaromi.tamas@semmelweis.hu (T.K.); rozsomd@hotmail.com (Z.R.); palmerperik@gmail.com (E.P.); povlivka.lorinc@semmelweis.hu (L.P.); bakos.regina@semmelweis-univ.hu (R.B.); szalai.borbala@semmelweis.hu (B.S.); muller.veronika@semmelweis.hu (V.M.); 2 Institue of Preventive Medicine and Public Health, Semmelweis University, 1085 Budapest, Hungary; fekete.monika@semmelweis.hu; 3 Fonyod Health Center, 8640 Fonyod, Hungary; gszendro@gmail.com

**Keywords:** COPD, sarcopenia, frailty, pulmonary rehabilitation, exercise therapy, nutritional supplementation, pharmacological interventions, multimodal therapy

## Abstract

Introduction: Sarcopenia and frailty are prevalent yet under-recognized contributors to disability, impaired quality of life, and adverse outcomes in chronic obstructive pulmonary disease (COPD). Shared mechanisms, including systemic inflammation, hormonal dysregulation, malnutrition, and physical inactivity, render these syndromes important targets for multimodal intervention. This review summarizes current evidence on exercise-based, nutritional, pharmacological, and adjunctive strategies for their management in COPD. Materials and Methods: This narrative review is based on a structured literature search of PubMed, Scopus, and Embase to identify relevant studies published between January 2000 and May 2025. Eligible publications included randomized controlled trials, meta-analyses, systematic reviews, and observational studies involving adults with COPD and documented sarcopenia and/or frailty. Interventions were categorized by modality, and outcomes included muscle mass, strength, physical performance, quality of life, and hospitalizations. Data were synthesized thematically. Results: Resistance and combined exercise training consistently improved muscle strength and physical function, while endurance training enhanced cardiorespiratory capacity, particularly within pulmonary rehabilitation programs. Nutritional interventions, especially protein, leucine, or β-hydroxy-β-methylbutyrate supplementation, supported gains in lean mass and exercise tolerance. Pharmacological strategies, including anabolic hormones and myostatin inhibitors, showed early promise but require further evaluation regarding safety and long-term efficacy. Adjunctive therapies, such as neuromuscular electrical stimulation and oxygen supplementation, benefited patients unable to participate in conventional exercise training. Conclusions: An integrated, multimodal approach combining structured exercise training and targeted nutritional support should be considered a cornerstone of COPD management to prevent and treat sarcopenia and frailty. Personalized rehabilitation strategies can substantially improve functional outcomes and quality of life, while future research should prioritize biomarker-guided personalization and long-term intervention studies.

## 1. Introduction

Chronic obstructive pulmonary disease (COPD) is a progressive respiratory condition characterized by persistent airflow limitation and chronic systemic inflammation [1,2]. While primarily affecting the lungs, COPD is increasingly recognized as a systemic disorder with significant extra-pulmonary manifestations [3,4]. Among the most debilitating of these are sarcopenia, defined as the progressive and generalized loss of skeletal muscle mass and strength, and frailty, a multi-dimensional syndrome marked by reduced physiological reserve and increased vulnerability to stressors [5,6].

According to the revised European Working Group on Sarcopenia in Older People (EWGSOP2) consensus, sarcopenia is diagnosed based on the presence of low muscle strength as the primary parameter, confirmed by low muscle quantity or quality, and further characterized by impaired physical performance to define severity [6,7]. Diagnostic assessment typically involves handgrip strength, chair stand tests, dual-energy X-ray absorptiometry (DXA), bioelectrical impedance analysis (BIA), and functional performance measures such as gait speed or the Short Physical Performance Battery (SPPB) [8]. In contrast, frailty is conceptualized as a broader clinical syndrome encompassing physical, psychological, cognitive, and social vulnerability [9]. Common diagnostic frameworks include the Fried frailty phenotype, which focuses on physical components such as weakness, slowness, exhaustion, weight loss, and low physical activity, and the Rockwood frailty index, which operationalizes frailty as the accumulation of health deficits across multiple domains [10,11]. Thus, while sarcopenia primarily reflects skeletal muscle impairment, frailty represents a multidimensional vulnerability state, of which sarcopenia constitutes a central but not exclusive component [10,12].

These two conditions often coexist in COPD patients, particularly in advanced stages of the disease, and contribute significantly to adverse health outcomes [1,8,10]. Their overlap reflects shared underlying biological mechanisms, yet their partial independence underscores the importance of parallel but distinct diagnostic and therapeutic strategies.

The burden of sarcopenia and frailty on COPD outcomes is substantial [13]. Both are independently associated with decreased exercise capacity, greater symptom burden, impaired health-related quality of life, increased frequency and severity of exacerbations, longer hospital stays, higher rates of institutionalization, and increased all-cause mortality [8,10,14]. Their presence compounds the effects of respiratory dysfunction, accelerating disease progression and complicating clinical management. Despite their profound impact, sarcopenia and frailty are frequently underdiagnosed and undertreated in routine COPD care [3,4,15].

Several COPD-specific mechanisms contribute to the development of sarcopenia and frailty [16,17]. Chronic systemic inflammation, driven by persistent airway irritation and recurrent exacerbations, promotes proteolysis and suppresses anabolic signaling [18,19]. Hypoxemia and oxidative stress impair mitochondrial function and muscle bioenergetics, while endocrine alterations, including testosterone deficiency and insulin resistance, further compromise muscle homeostasis [20,21]. In parallel, physical inactivity due to dyspnea, fatigue, and fear of exertion accelerates deconditioning, whereas nutritional insufficiency, anorexia, and altered metabolism exacerbate negative protein balance [22,23,24]. Collectively, these interrelated processes establish a vicious cycle of muscle wasting, functional decline, and escalating vulnerability.

The pathophysiology of these conditions in the context of COPD is therefore complex and multifactorial. Importantly, sarcopenia and frailty also amplify respiratory impairment by weakening ventilatory muscles, reducing exercise tolerance, impairing cough effectiveness, and increasing susceptibility to infections and exacerbations, thereby directly contributing to disease instability and healthcare utilization [25,26]. These systemic factors interact with behavioral and social determinants of health, creating a vicious cycle of functional decline and increased healthcare utilization.

Given this complexity, early and effective interventions are critical to alter the trajectory of sarcopenia and frailty in COPD [27]. Intervening at an early stage may help preserve muscle function, enhance physical resilience, and ultimately reduce the risk of adverse outcomes [28]. However, due to the multifactorial nature of these conditions, a single-modality treatment is unlikely to be sufficient. Instead, growing and increasingly robust clinical evidence supports the need for a multimodal therapeutic approach, integrating exercise training, nutritional support, pharmacologic agents, and adjunctive modalities such as oxygen therapy and tele-rehabilitation [29,30,31,32,33,34,35]. These interventions must be personalized and sustained to be effective in real-world clinical settings. This narrative review aims to provide a comprehensive overview of current and emerging therapeutic interventions targeting sarcopenia and frailty in patients with COPD.

### Objectives

The primary aim of this review is to critically evaluate current evidence on the role of exercise and nutritional interventions in the prevention and management of sarcopenia and frailty in patients with COPD. Specifically, we aim to:

- Synthesize mechanistic insights linking COPD-related pathophysiology to skeletal muscle dysfunction and systemic frailty;
- Critically assess the effectiveness of different exercise modalities and nutritional strategies, both as standalone and combined interventions;
- Identify key knowledge gaps and methodological limitations in the existing literature; and
- Propose clinically relevant perspectives for integrated, personalized intervention strategies targeting muscle health and functional capacity in COPD.

By adopting an integrative and translational perspective, this review seeks to provide practical guidance for clinicians and researchers and to highlight future directions for improving functional outcomes and quality of life in this vulnerable patient population.

## 2. Materials and Methods

### 2.1. Design and Conceptual Framework of the Review

This article was designed as a narrative, integrative review aimed at synthesizing and critically evaluating the current and emerging evidence on therapeutic strategies targeting sarcopenia and frailty in patients with COPD. Rather than applying the rigid methodological framework of a formal systematic review, we adopted a broad, hypothesis-generating and translational perspective, allowing conceptual integration across heterogeneous study designs, intervention modalities, and mechanistic domains. This narrative approach was selected to enable a comprehensive evaluation of complex, multifactorial interventions, facilitate clinical interpretation, and identify emerging therapeutic concepts and research gaps relevant to personalized COPD care.

### 2.2. Search Strategy and Data Sources

A comprehensive literature search was conducted to identify relevant studies examining interventions targeting sarcopenia and frailty in patients with chronic obstructive pulmonary disease. The electronic databases PubMed, Scopus, and Embase were systematically searched for peer-reviewed articles published between January 2000 and May 2025. The search strategy combined controlled vocabulary (MeSH terms) and free-text keywords, including: “COPD,” “chronic obstructive pulmonary disease,” “sarcopenia,” “frailty,” “muscle wasting,” “rehabilitation,” “exercise therapy,” “nutritional supplementation,” “pharmacologic therapy,” “tele-rehabilitation,” “neuromuscular electrical stimulation,” and “multimodal intervention,” used in various combinations. Boolean operators (“AND,” “OR”) were applied to optimize sensitivity and specificity. Search results were limited to studies published in English and involving human participants. In addition, reference lists of eligible studies and relevant review articles were manually screened to identify additional pertinent publications.

### 2.3. Eligibility Criteria

Studies were considered eligible if they met the following criteria: (i) adult participants (≥18 years) with a clinical diagnosis of COPD; (ii) documented assessment of sarcopenia and/or frailty based on established diagnostic criteria; and (iii) evaluation of therapeutic interventions targeting these conditions. Eligible study designs included randomized controlled trials, non-randomized controlled studies, observational cohort and case–control studies, and high-quality systematic reviews and meta-analyses. Primary outcomes of interest were changes in muscle mass, muscle strength, physical performance, and frailty status. Secondary outcomes included quality of life, exercise capacity, hospitalization rates, exacerbation frequency, and mortality.

Studies were excluded if they: (i) did not explicitly assess sarcopenia or frailty; (ii) involved pediatric populations; (iii) were case reports, conference abstracts, editorials, or narrative commentaries without original data; or (iv) lacked peer review.

### 2.4. Data Extraction and Narrative Synthesis

Data extraction was performed independently by two reviewers using a standardized data collection framework. Extracted information included study design, population characteristics, diagnostic definitions, intervention modalities, outcome measures, and principal findings. Discrepancies were resolved through discussion and consensus.

Given the substantial heterogeneity in study populations, interventions, and outcome definitions, a narrative synthesis approach was adopted rather than quantitative meta-analysis. Studies were grouped thematically according to intervention categories, and findings were synthesized with emphasis on clinical applicability, mechanistic insights, comparative effectiveness, and translational relevance.

### 2.5. Study Selection Process and Flow Diagram

**Figure 1:**
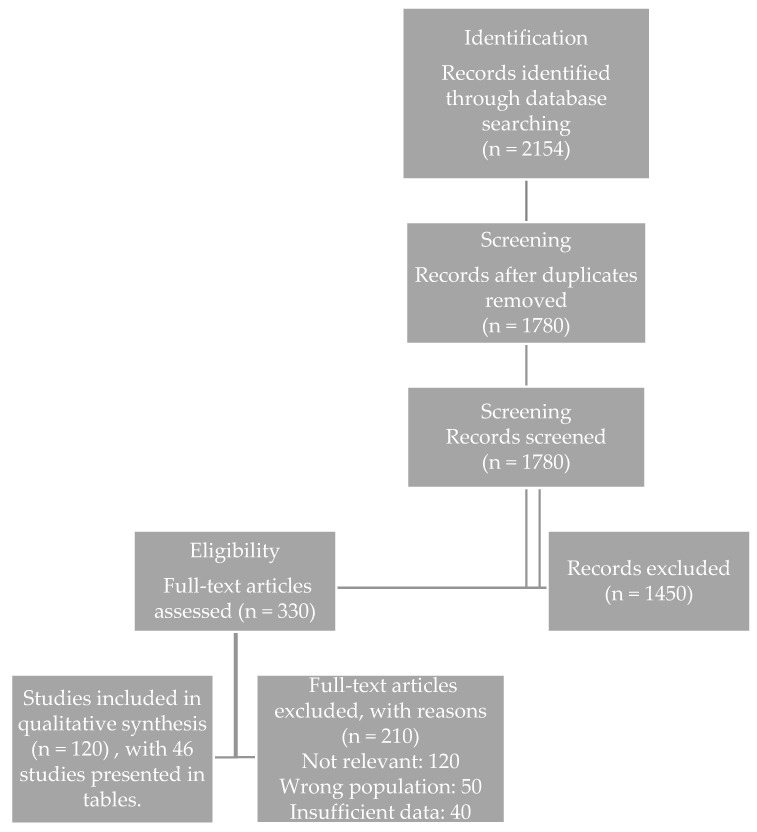
PRISMA flow diagram illustrating the selection process for studies included in the review of interventions targeting sarcopenia and frailty in COPD.

The study selection process followed a structured narrative approach to ensure transparency and comprehensive coverage of the relevant literature. Records identified through database searches were initially screened based on titles and abstracts, followed by full-text evaluation of potentially relevant publications. Studies meeting the predefined thematic and methodological criteria were subsequently included in the narrative synthesis. The overall workflow of the literature selection process is summarized in the flow diagram (Figure 1).

## 3. Results

This narrative review includes a total of 120 relevant publications, comprising systematic and narrative reviews as well as original clinical studies. Of these, 46 studies are presented in detail in tabulated form to provide a structured comparison of the most important intervention strategies, study designs, and clinical outcomes. The results are organized thematically according to the major intervention domains. In addition, selected clinical data from our pulmonary rehabilitation cohort are presented for illustrative purposes to contextualize the reviewed evidence and to highlight the real-world relevance of body composition assessment in COPD management.

### Principles of Management

Managing sarcopenia and frailty in COPD requires a shift from reactive treatment to a proactive, personalized, and multidisciplinary approach [36]. Given the complex and multifactorial nature of these conditions, intervention must be comprehensive and initiated early to be most effective. Multidisciplinary teams—including pulmonologists, physiotherapists, nutritionists, geriatricians, and mental health professionals—work collaboratively to address the wide-ranging physiological, nutritional, and psychosocial needs of patients, ultimately improving outcomes and ensuring continuity of care [37]. Personalized strategies, guided by thorough assessments of physical function, nutritional status, and, where available, emerging biomarkers, help tailor interventions to each patient’s specific disease severity and comorbidities [38]. Initiating treatment during the pre-frail stage offers the best chance of reversing or slowing progression, whereas late-stage management focuses on maintaining function and reducing complications. Incorporating routine screening and, in the future, digital health tools may further support early detection and timely, targeted intervention [39].

## 4. Exercise-Based Interventions

Exercise training is a fundamental therapeutic approach for managing sarcopenia and frailty in COPD, as it mitigates muscle wasting, enhances exercise tolerance, and reduces the risk of exacerbations [17]. Pulmonary rehabilitation (PR) represents the most comprehensive framework, combining structured exercise with education, nutritional support, and psychosocial care [14,40,41]. This multidimensional strategy not only improves pulmonary outcomes but also directly addresses systemic impairments contributing to sarcopenia and frailty [14,40,41]. Despite robust evidence of its efficacy, PR is frequently underutilized due to barriers such as limited accessibility, comorbidities, and poor adherence, highlighting the importance of individualized and flexible program designs [42].

### 4.1. Resistance Training

Resistance training (RT) is the most effective intervention for increasing muscle mass and strength in sarcopenic patients with COPD [43]. By promoting hypertrophy through mechanical overload and anabolic signaling, RT enhances quadriceps size, walking distance, and daily functional performance [44]. Compared with endurance modalities, it produces greater improvements in muscle strength and reduces fall risk, though implementation requires access to equipment, supervision, and progressive load adjustments [45]. These demands may pose challenges for severely frail individuals, but when carefully individualized, RT consistently yields substantial functional benefits, making it a cornerstone of rehabilitation strategies.

### 4.2. Endurance and Aerobic Training

Endurance training targets cardiovascular fitness, oxidative metabolism, and ventilatory efficiency, contributing to improved exercise tolerance, dyspnea management, and quality of life [46]. While its impact on muscle hypertrophy is limited, endurance training remains accessible and well-integrated into PR. Interval training, involving alternating bouts of higher-intensity activity with recovery, is particularly valuable for patients with severe dyspnea or poor exercise tolerance [47]. It improves walking distance and aerobic capacity with reduced perceived exertion compared to continuous training, though it requires close monitoring to ensure both safety and adherence [48].

### 4.3. Pulmonary Rehabilitation Programs

Comprehensive PR programs combine resistance and endurance exercise with education, nutritional guidance, and psychosocial support, addressing both pulmonary limitations and systemic contributors to frailty and sarcopenia [49]. Evidence indicates that sarcopenic patients achieve outcomes comparable to, or even exceeding, those without sarcopenia when enrolled in PR [50]. Benefits include improved frailty indices, muscle strength, exercise capacity, and health-related quality of life. Nonetheless, real-world effectiveness is hindered by barriers such as transportation difficulties, comorbidities, and low completion rates. Program adherence remains the strongest predictor of long-term outcomes, underscoring the importance of strategies that support sustained participation [51].

To illustrate the clinical relevance and real-world applicability of body composition assessment in pulmonary rehabilitation, we present representative baseline data from 352 COPD patients participating in our institutional PR program (Table 1).

This cohort demonstrates a high prevalence of increased adiposity, reduced relative skeletal muscle mass, and elevated visceral fat, underscoring the frequent coexistence of sarcopenic obesity and metabolic dysregulation in COPD. These findings reinforce the importance of integrating targeted nutritional strategies and quality-of-life interventions alongside exercise training to optimize functional recovery, enhance exercise tolerance, and improve long-term prognosis. Importantly, these data are provided for contextual and illustrative purposes and are not intended as a primary outcome analysis.

### 4.4. Home-Based and Tele-Rehabilitation Approaches

Home-based and tele-rehabilitation (tele-PR) programs have emerged as practical alternatives to traditional center-based rehabilitation, particularly for patients with mobility limitations or geographic barriers [34,52]. These approaches, supported by digital platforms, wearable devices, and remote supervision, have demonstrated efficacy comparable to conventional PR in improving the six-minute walk distance, dyspnea, and quality of life [34,52]. However, their success is contingent upon patient motivation, digital literacy, and access to reliable technology. Novel strategies, including caregiver involvement, behavior-change interventions, and artificial intelligence-assisted exercise prescription, show promise in improving adherence, though evidence regarding long-term sustainability and clinical outcomes remains limited [53].

### 4.5. Indications and Clinical Rationale for Neuromuscular Electrical Stimulation

Neuromuscular electrical stimulation (NMES) represents a passive training modality for patients with advanced frailty or severe exercise intolerance who are unable to participate in conventional exercise programs. By eliciting muscle contractions through externally applied electrical impulses, NMES may improve muscle strength, endurance, and tolerance to physical activity, thereby serving as a valuable adjunct to comprehensive rehabilitation strategies [35]. Although its effects on peak power output and muscle hypertrophy appear less consistent than those achieved with traditional resistance training, NMES has demonstrated clinically meaningful improvements in functional status and quality of life among highly deconditioned individuals [54]. Accordingly, integration of NMES into multimodal rehabilitation frameworks may facilitate broader access to exercise-based interventions in the most vulnerable COPD populations [55].

### 4.6. Resistance and Endurance Training

Endurance and resistance training improve physiological parameters through complementary mechanisms [56]. Resistance training primarily enhances muscle strength, muscle mass, frailty status, and quality of life, whereas endurance training mainly improves cardiovascular fitness, metabolic efficiency, frailty indices, and lung mechanics, thereby influencing exercise tolerance, oxygen uptake, dyspnea, and health-related quality of life [45]. Combining both modalities within structured, individualized rehabilitation programs allows for synergistic benefits, addressing both peripheral muscle dysfunction and cardiopulmonary limitations, and represents the optimal strategy for comprehensive functional restoration in COPD [57]. Collectively, these findings support a phenotype-driven, multimodal rehabilitation paradigm in COPD, where intervention selection is guided by baseline muscle mass, functional capacity, frailty status, and comorbidity burden. Key exercise-based interventions targeting sarcopenia and frailty in COPD, including resistance and endurance training, pulmonary rehabilitation, home-based programs, and NMES, are summarized in Table 2.

## 5. Nutritional Interventions

Malnutrition, sarcopenia, and frailty commonly co-occur in COPD due to systemic inflammation, hypermetabolism, and impaired protein turnover, all of which accelerate muscle catabolism, impair functional capacity, and worsen clinical outcomes [73,74]. Disease-related undernutrition further exacerbates sarcopenia, highlighting the importance of tailored nutritional strategies to preserve muscle mass and function [75].

### 5.1. Protein and Amino Acid Supplementation

Adequate protein intake, generally recommended at 1.2–2.0 g/kg/day, is essential to preserve muscle mass in COPD, particularly when combined with exercise training [76]. Supplementation with leucine, leucine-enriched essential amino acids, or β-hydroxy β-methylbutyrate (HMB) enhances anabolic signaling through mTOR activation and has been shown to improve lean body mass and strength [33,74,77]. Optimizing timing and distribution, particularly with post-exercise intake, further augments the anabolic response. Nevertheless, anabolic resistance in older adults and individuals with advanced COPD may blunt the full benefits, underscoring the need for higher-quality or specifically enriched protein formulations [78].

### 5.2. Caloric Optimization and Energy Balance

Because patients with COPD often experience a 10–15% increase in energy requirements due to hyper-metabolism and catabolic stress, nutritional interventions must prioritize energy-dense, protein-rich diets [79]. Oral nutritional supplements (ONS) enriched with protein, vitamins, and minerals have consistently improved body weight, lean mass, and physical function in undernourished patients (Table 3). Macronutrient distribution should emphasize high-quality protein, balanced carbohydrate intake to minimize CO_2_ production, and healthy fats to ensure caloric adequacy. Adherence to these regimens may be challenging due to appetite loss, early satiety, and dyspnea during meals, but targeted strategies can mitigate these barriers [80].

### 5.3. Micronutrient Support

Specific micronutrients also play critical roles in muscle health and frailty prevention. Vitamin D deficiency is highly prevalent in COPD and is associated with impaired muscle function and increased exacerbation risk; supplementation with 800–2000 IU/day is recommended in deficient individuals, though benefits appear limited to those with baseline deficiency [29,31,85,86]. Omega-3 polyunsaturated fatty acids (EPA and DHA) exert anti-inflammatory and anabolic effects, supporting muscle preservation and exercise capacity, especially when combined with exercise [87,88,89,90]. Antioxidant micronutrients such as vitamins C and E and selenium may reduce oxidative stress, though their impact on sarcopenia outcomes remains inconclusive, reflecting heterogeneity in trial results (Table 3).

### 5.4. Delivery Models and Challenges

Optimal delivery of nutritional interventions requires individualized dietary counseling, ideally provided by registered dietitians [33,91,92]. Counseling should address macronutrient balance, micronutrient adequacy, meal timing, and appropriate use of oral nutritional supplements [93]. Multidisciplinary management that integrates nutritional strategies with exercise and psychosocial support is essential to improve adherence and achieve sustainable benefits [94]. Barriers such as socioeconomic constraints, limited service access, appetite loss, and poor tolerance of supplements often undermine outcomes, highlighting the need for personalized, flexible, and long-term approaches.

## 6. Pharmacological and Experimental Therapies

In advanced COPD or in patients unable to engage in exercise or nutritional programs, pharmacological and experimental interventions provide potential adjunctive or alternative strategies [95]. These approaches target mechanisms underlying muscle wasting, including hormonal imbalance, chronic inflammation, and impaired myogenesis [96]. The main therapeutic domains encompass anabolic hormone therapy, anti-inflammatory and metabolic agents, myostatin inhibition, and respiratory-targeted interventions with indirect benefits [96,97].

### 6.1. Anabolic Hormone Therapy

Testosterone and other androgens stimulate muscle protein synthesis, satellite cell activation, and neuromuscular function. Clinical trials in COPD show modest improvements in lean mass, muscle strength, and exercise capacity, particularly when testosterone is combined with training [98]. However, adverse events—including erythrocytosis, cardiovascular complications, and prostate-related risks—restrict its use to hypogonadal patients under specialist monitoring. Growth hormone (GH) acting through the IGF-1 pathway, can promote muscle regeneration but produces inconsistent functional benefits and frequently causes side effects such as glucose intolerance, insulin resistance, and edema [27]. Selective androgen receptor modulators (SARMs), such as enobosarm, provide a more targeted anabolic effect with reduced androgenic toxicity. Early studies in catabolic conditions demonstrate increased muscle mass, though COPD-specific data remain sparse. Collectively, anabolic hormone strategies show potential for enhancing lean body mass, but their effects on functional outcomes are mixed, and safety concerns limit their widespread adoption [47,98].

### 6.2. Anti-Inflammatory and Metabolic Agents

Chronic systemic inflammation in COPD drives catabolism through cytokine-mediated protein degradation and mitochondrial dysfunction [99]. Several pharmacological agents target these pathways. Statins exert anti-inflammatory effects and may attenuate cytokine-driven muscle breakdown; however, their benefit in sarcopenia is inconsistent, and the risk of myopathy limits applicability [100]. Metformin, via AMPK activation, enhances mitochondrial biogenesis and reduces systemic inflammation [101]. Preliminary findings suggest it may help preserve muscle mass and function in older adults, though its role in COPD remains under investigation. Anti-cytokine therapies, such as TNF-α inhibitors, theoretically mitigate cachexia but have limited clinical data in COPD and carry substantial infection risks [102]. Thus, while anti-inflammatory and metabolic therapies represent mechanistically appealing interventions, their current evidence base is insufficient for routine application [100].

### 6.3. Myostatin Inhibitors and Emerging Molecules

The myostatin signaling pathway negatively regulates muscle growth and is upregulated in COPD-associated sarcopenia [103]. Pharmacological inhibition of this pathway has attracted considerable attention. Bimagrumab, an anti-ActRIIB monoclonal antibody, significantly increases lean mass in phase II trials, yet functional improvements such as strength and exercise capacity have been minimal, emphasizing the necessity of pairing pharmacotherapy with exercise-based rehabilitation [104,105]. Other experimental strategies include agents such as espindolol, which combines anabolic and β-blocking effects, follistatin-based gene therapies to enhance hypertrophy, and mitochondrial-targeted antioxidants to combat oxidative stress [103]. While promising in preclinical and early-phase studies, these agents remain unapproved, and their long-term safety and efficacy in COPD are unproven [104,105].

### 6.4. Respiratory-Targeted Therapies with Indirect Benefits

Although not primarily developed for sarcopenia or frailty, pharmacological and procedural interventions targeting pulmonary function may indirectly preserve skeletal muscle by improving ventilatory efficiency, reducing dyspnea, and enhancing physical activity tolerance. Long-acting bronchodilators, including LABAs (e.g., salmeterol) and LAMAs (e.g., tiotropium), reduce dynamic hyperinflation and improve exertional tolerance [106]. Evidence consistently shows that they facilitate participation in rehabilitation and support functional improvements, though they exert minimal direct anabolic effects [106]. Inhaled corticosteroids, particularly when combined with LABAs, decrease airway inflammation but have limited systemic impact on muscle preservation; unlike systemic corticosteroids, they carry a lower risk of myopathy [107]. Lung volume reduction interventions—surgical or bronchoscopic—restore diaphragmatic function, reduce hyperinflation, and alleviate exertional dyspnea. Trials, including the National Emphysema Treatment Trial, confirm improvements in quality of life and functional outcomes, though risks and strict selection criteria limit eligibility [108]. Long-term oxygen therapy (LTOT) improves survival in hypoxemic COPD and may reduce oxidative stress–mediated wasting, whereas non-invasive ventilation (NIV) reduces respiratory workload, facilitates sleep, and allows higher-intensity rehabilitation, modalities support muscle preservation indirectly but face challenges related to adherence and patient selection [109,110,111] (Table 4).

## 7. Non-Pharmacologic and Adjunctive Therapies

Non-pharmacologic interventions are essential in the management of sarcopenia and frailty in COPD, especially for patients unable to participate in exercise-based or pharmacological pro-grams. These approaches aim to preserve independence, optimize functional capacity, and ad-dress physical, cognitive, psychological, and social dimensions, either as stand-alone strategies or within comprehensive rehabilitation frameworks [127].

### 7.1. Practical Implementation and Protocol Considerations for Neuromuscular Electrical Stimulation

Neuromuscular electrical stimulation applies low-frequency electrical currents to induce involuntary muscle contractions and constitutes a practical therapeutic option for deconditioned patients with COPD who are unable to engage in conventional exercise training. Evidence from clinical trials and meta-analyses indicates improvements in quadriceps strength, muscle mass, endurance, mobility, and quality of life following NMES interventions [35]. Standard protocols typically involve stimulation frequencies of 35–50 Hz, pulse widths of 200–400 µs, and session durations of 20–60 min, administered five to seven days per week over a period of four to six weeks. Despite these benefits, NMES use may be limited by discomfort, equipment dependency, and interindividual variability in responsiveness, underscoring the need for supervision and individualized parameter adjustment [64].

### 7.2. Oxygen Therapy in Frail COPD Patients

Long-term oxygen therapy plays a critical role in patients with hypoxemia (PaO_2_ < 55 mmHg or SpO_2_ < 88%) where it improves oxygen delivery, reduces oxidative stress, and enhances exercise tolerance while lowering fatigue and hospitalization risk [128]. These benefits are, however, restricted to hypoxemic individuals; in non-hypoxemic patients, oxygen supplementation does not improve outcomes and may even discourage physical activity. Careful patient selection is therefore essential to optimize its impact [129].

### 7.3. Cognitive, Psychological, and Social Support

The psychosocial dimensions of frailty are equally important, as cognitive decline, depression, and social isolation are common in COPD and substantially impair functional outcomes and adherence to therapy [130]. Interventions such as cognitive-behavioral therapy, peer-support groups, and occupational therapy have been shown to enhance motivation, improve coping, and reduce depressive symptoms. Social support also facilitates smoking cessation, which remains a corner-stone intervention by reducing oxidative damage, systemic inflammation, and skeletal muscle loss [16]. However, stigma, limited access to services, and high relapse rates pose challenges, under-scoring the need for structured, multidisciplinary support programs.

### 7.4. Fall Prevention and Functional Independence Training

Frailty substantially increases the risk of falls in COPD due to impaired balance, muscle weak-ness, and reduced proprioception. Fall prevention programs integrating balance and gait training, environmental modifications, and dual-task exercises are effective in mitigating this risk [13]. Functional independence training further promotes autonomy, reduces hospitalization, and enhances overall quality of life, with the best outcomes observed when delivered as part of multidisciplinary pulmonary rehabilitation [131].

## 8. Integrated Multimodal Approaches

The multifactorial pathophysiology of sarcopenia and frailty in COPD necessitates integrated interventions combining exercise, nutritional optimization, pharmacologic support, and psycho-social care [132,133,134]. Multimodal strategies yield superior improvements in muscle function, hospitalization rates, and quality of life compared to isolated approaches [135,136].

### 8.1. Synergistic Effects of Combined Interventions

Exercise and nutritional support exert synergistic anabolic effects, particularly in the catabolic –inflammatory context of COPD [137]. Resistance training enhances muscle protein synthesis via mechanotransduction, further potentiated by adequate protein or leucine-enriched supplementation [76]. Adjunctive anabolic agents, such as testosterone, may augment gains in muscle mass and strength, though benefits must be balanced against potential risks [98].

### 8.2. Real-World Programs and Pilot Studies

Pilot studies and real-world programs integrating multimodal interventions have demonstrated clinically meaningful benefits in patients with COPD. Multicomponent strategies combining structured exercise training, high-protein nutritional supplementation, and individualized counseling have been associated with improvements in gait speed, handgrip strength, and overall physical performance, as assessed by validated functional indices such as the Short Physical Performance Battery [78]. In addition, integrated care models incorporating pulmonary rehabilitation, optimized pharmacotherapy, and structured home-based resistance training programs have been shown to reduce hospital readmissions and improve functional independence over medium-term follow-up. Telehealth-supported rehabilitation approaches, combining remote exercise supervision, nutritional monitoring, and psychosocial support, have achieved outcomes comparable to conventional center-based programs, while improving accessibility, adherence, and continuity of care in real-world clinical settings [138].

### 8.3. Models of Care and Delivery Pathways

Optimal multimodal delivery relies on coordinated, interdisciplinary frameworks. Pulmonary rehabilitation centers function as integration hubs, supported by physiotherapists, dietitians, psychologists, and respiratory specialists. Community-based and telehealth models enhance accessibility for frail or remote patients, allowing personalized, data-driven adjustments and ensuring adherence, safety, and continuity of care in both routine and post-pandemic contexts [139,140].

## 9. Discussion

Sarcopenia and frailty represent two interconnected geriatric syndromes that substantially contribute to disease burden, disability, and adverse outcomes in patients with COPD. Although often unrecognized in clinical practice, these conditions are now established as major contributors to disability, hospitalizations, and mortality in COPD populations. Beyond their shared clinical consequences, sarcopenia and frailty represent distinct but overlapping constructs: sarcopenia primarily reflects progressive loss of skeletal muscle mass and strength, whereas frailty encompasses a multidimensional vulnerability state involving physical, cognitive, psychological, and social domains. Their frequent coexistence in COPD underscores the need for integrated assessment frameworks and targeted interventions addressing both muscular and systemic components of vulnerability [25].

Current evidence highlights a range of interventions—including exercise, nutritional support, pharmacologic therapies, and non-pharmacologic adjuncts—with varying degrees of efficacy. Among these, exercise-based therapies, particularly resistance and multicomponent training, demonstrate the most consistent benefits in improving muscle mass, strength, and functional capacity. Importantly, multimodal rehabilitation programs combining resistance, endurance, and balance training appear to exert synergistic effects, improving not only physical performance but also frailty indices and overall resilience, particularly when delivered within structured pulmonary rehabilitation frameworks [141,142].

However, feasibility may be limited in advanced COPD due to dyspnea, fatigue, or comorbidities, necessitating alternative approaches such as neuromuscular electrical stimulation. Nutritional interventions, including adequate protein intake (1.2–2.0 g/kg/day) and leucine-enriched or omega-3-supplemented formulations, show promise in enhancing anabolic responses, particularly when combined with exercise. Emerging evidence further suggests that individualized nutritional strategies guided by baseline nutritional status, inflammatory burden, and metabolic phenotype may optimize therapeutic responses, highlighting the importance of precision-based nutritional care [33].

Yet their implementation is often hindered by reduced appetite, gastrointestinal intolerance, or socioeconomic barriers. Pharmacologic agents such as testosterone, selective androgen receptor modulators, and myostatin inhibitors remain investigational, with inconsistent translation of muscle mass gains into functional improvements. This discrepancy emphasizes that pharmacologically induced hypertrophy alone is insufficient and must be integrated with functional training to achieve clinically meaningful outcomes [143].

Additionally, non-pharmacologic strategies—including long-term oxygen therapy, psychosocial support, and fall prevention—address broader aspects of frailty, reinforcing the need for a holistic, biopsychosocial approach. Cognitive, emotional, and social determinants substantially influence adherence, functional recovery, and long-term prognosis, underscoring the necessity of multidisciplinary care models extending beyond conventional respiratory management [144,145].

Despite these advances, key limitations persist, including diagnostic heterogeneity, short intervention durations, and underrepresentation of high-risk subgroups. Moving forward, a multi-modal, patient-centered model—incorporating risk stratification, tiered interventions, and regular reassessment—is essential [146]. Future research should prioritize longitudinal studies, biomarker-guided therapies, and innovative delivery models (e.g., tele-rehabilitation, wearable sensors) to optimize personalized care. Furthermore, the integration of digital health tools and artificial intelligence–driven monitoring platforms may facilitate early detection of functional decline and dynamic adjustment of interventions, potentially improving long-term adherence and clinical outcomes. Ultimately, addressing sarcopenia and frailty in COPD requires integrated, multidisciplinary strategies to improve resilience and preserve autonomy in this vulnerable population.

## 10. Clinical Implications and Future Research Priorities

Based on the integrated evaluation of current evidence, several clinically relevant implications emerge. First, systematic screening for sarcopenia and frailty should be incorporated into routine COPD care, particularly in patients with advanced disease, frequent exacerbations, reduced exercise tolerance, or unintentional weight loss. Early identification may facilitate timely intervention, prevent irreversible functional decline, and improve long-term outcomes.

Second, multimodal, individualized intervention strategies should be prioritized, integrating structured exercise training, targeted nutritional support, and selected pharmacological or adjunctive therapies. Tailoring interventions according to disease severity, functional status, comorbidity burden, and patient preferences enables a precision rehabilitation approach, maximizing adherence and clinical benefit.

Future research should prioritize:

- the development and validation of biomarker-guided risk stratification tools for early detection and monitoring of sarcopenia and frailty in COPD;
- long-term, adequately powered randomized controlled trials evaluating combined exercise–nutrition–pharmacological interventions with clinically meaningful endpoints;
- implementation and health-services research addressing feasibility, cost-effectiveness, and scalability in real-world clinical settings; and
- digital health and tele-rehabilitation strategies to improve accessibility, long-term adherence, and continuity of care, particularly in underserved populations.

## 11. Conclusions

Sarcopenia and frailty are key, yet frequently under-recognized determinants of poor outcomes in COPD, strongly associated with functional decline, reduced quality of life, hospitalizations, and mortality. Their complex pathophysiology requires integrated, multimodal, and individualized management strategies combining structured exercise, targeted nutrition, and selected adjunctive therapies. Coordinated interventions provide superior clinical benefits, including improved muscle function, enhanced physical performance, and reduced healthcare utilization. Integrating systematic screening and personalized rehabilitation strategies into routine COPD care may therefore represent a crucial opportunity to preserve functional independence and improve long-term patient-centered outcomes.

## Figures and Tables

**Table 1:** Body composition parameters in COPD patients (n = 352) before pulmonary rehabilitation.

Parameter	Mean ± SD
Height (cm)	167.5 ± 14.9
Weight (kg)	79.8 ± 22.8
Body Mass Index (kg/m^2^)	28.1 ± 6.9
Body fat (%)	34.3 ± 10.8
Skeletal muscle (%)	27.7 ± 5.4
Resting metabolic rate (kcal)	1611.8 ± 316.9
Visceral fat (index)	11.3 ± 5.5

Values are expressed as mean ± standard deviation. Data represent COPD patients who participated in the pulmonary rehabilitation program.

**Table 2:** Exercise-Based Interventions for Sarcopenia and Frailty in COPD: Evidence from Clinical Trials.

Author, Year	Study Design/Population	Intervention Category	Intervention Details	Comparator/Control	Outcome Measures	Key Findings	Limitations
Bernard et al., 1999 [58]	RCT, COPD patients	Resistance Training	12-wk supervised RT (3×/wk, progressive loads)	Usual care	Quadriceps CSA, 6MWD, strength	↑ Muscle strength, CSA, and 6MWD	Small sample; short follow-up
Spruit et al., 2002 [59]	RCT, severe COPD	Resistance Training	RT vs endurance training	Endurance training	Muscle strength, exercise tolerance	RT superior in strength; endurance ↑ VO_2_peak	No combined arm
Probst et al., 2011 [60]	RCT, moderate-severe COPD	Resistance Training	12-wk RT with elastic bands	Usual care	Handgrip, sit-to-stand, HRQoL	Improved functional performance and QoL	Limited intensity progression
Maltais et al., 1997 [61]	RCT, moderate-severe COPD	Endurance Training	12-wk continuous training (cycling, 60–70% VO_2_max)	Usual care	Exercise capacity, dyspnea	↑ VO_2_max, ↓ dyspnea	No frailty-specific outcomes
Vogiatzis et al., 2002 [47]	RCT, severe COPD	Endurance Training	Interval vs continuous training	Continuous training	6MWD, dyspnea	Interval training improved tolerance with less dyspnea	Small sample
Puhan et al., 2006 [62]	RCT, COPD	Endurance Training	8-wk aerobic training (walking/cycling)	Standard care	HRQoL, 6MWD	↑ HRQoL, 6MWD	No muscle mass outcomes
Zwerink et al., 2014 [63]	Meta-analysis, COPD	Endurance Training	Aerobic training (8–24 wks)	Control	Dyspnea, 6MWD	Consistent ↑ tolerance, QoL	No hypertrophy effect
Spruit et al., 2013 [4]	Prospective cohort, COPD	Pulmonary Rehabilitation	12-wk multicomponent PR	Usual care	Exercise capacity, QoL, frailty	↑ 6MWD, QoL, frailty indices	Selection bias
Maddocks et al., 2016 [64]	RCT, COPD with frailty	Pulmonary Rehabilitation	8-wk PR	Usual care	Frailty phenotype, strength	Improved frailty scores, quadriceps strength	Short follow-up
Gloeckl et al., 2018 [65]	Review COPD	Pulmonary Rehabilitation	High-intensity PR vs standard PR	Standard PR	Exercise tolerance, QoL	Greater gains with high-intensity PR	Safety in severe frailty uncertain
McCarthy et al., 2015 [66]	Cochrane review	Pulmonary Rehabilitation	PR programs (varied protocols)	Usual care	HRQoL, exercise capacity	Strong evidence for PR effectiveness	Heterogeneity; adherence barriers
Maltais et al., 2008 [67]	RCT, COPD	Home/Tele-PR	8-wk home-based exercise program	Usual care	6MWD, dyspnea, HRQoL	Comparable to outpatient PR	Adherence variable
Holland et al., 2017 [34]	RCT, moderate-severe COPD	Home/Tele-PR	Tele-PR via video [8 wks]	Conventional PR	Exercise capacity, QoL	Non-inferior to center PR	Requires digital literacy
Bourne et al., 2017 [32]	RCT, COPD	Home/Tele-PR	Web-based PR (12 wks)	Usual care	Exercise tolerance, QoL	↑ 6MWD, QoL; lower completion than PR	High attrition
Cox et al., 2021 [68]	Systematic review	Home/Tele-PR	Home/tele-PR vs center PR	Center PR	Functional outcomes, QoL	Comparable short-term outcomes	Long-term sustainability uncertain
Neder et al., 2002 [69]	RCT, severe COPD	NMES	NMES (30 min/day, 6 wks)	Usual care	Strength, endurance	↑ Quadriceps endurance and strength	Small cohort
Vivodtzev et al., 2006 [70]	RCT, severe COPD	NMES	NMES vs sham	Sham	Exercise tolerance, QoL	↑ 6MWD, QoL, muscle strength	Short-term
Sillen et al., 2008 [71]	RCT, advanced COPD	NMES	NMES (5×/wk, 6 wks)	Usual care	Muscle mass, performance	Functional benefits, limited hypertrophy	Small sample
Zambom-Ferraresi et al., 2015 [72]	RCT; moderate–severe COPD	Resistance training	12-wk combined RT/(1×/wk) + endurance (1×/wk)	RT alone (2×/wk)	Strength, power, Wmax, 6MWD, QoL	Similar strength & 6MWD gains; ↑ muscle power and ↑ endurance with combined training	Small sample; short follow-up

COPD: chronic obstructive pulmonary disease; CSA: cross-sectional area; HRQoL: health-related quality of life; NMES: neuromuscular electrical stimulation; PR: pulmonary rehabilitation; QoL: quality of life; RCT: randomized controlled trial; RT: resistance training; VO_2_max: maximal oxygen uptake; Wmax: maximal workload; wk: week; 6MWD: 6-min walk distance; ↑: increase; ↓: decrease. Arrows indicate direction of change compared with the control or comparator group.

**Table 3:** Nutritional Strategies to Mitigate Sarcopenia and Frailty in COPD: Clinical Evidence.

Author, Year	Study Design/Population	Intervention Details	Comparator/Control	Outcome Measures	Key Findings	Limitations
Dal Negro et al., 2012 [22]	RCT; COPD with malnutrition	High-protein ONS (400–600 kcal/day, 12–20 g protein) for 12 wks	Standard diet	Body weight, FFM, 6MWD, QoL	↑ body weight, FFM, and exercise tolerance	Small sample size; adherence variable
Steiner et al., 2003 [77]	RCT; undernourished COPD	ONS + exercise training, 12 wks	Exercise alone	FFM, strength, 6MWD	Combined ONS + training ↑ muscle mass & functional gains more than training alone	High dropout; adherence issues
Broekhuizen et al., 2005 [73]	RCT; COPD, weight loss ≥ 5%	Nutrient-enriched ONS (protein, carbs, fat, vits/minerals) for 12 wks	Placebo ONS	Weight, muscle function, exercise capacity	↑ weight and functional status	Modest effect on muscle strength
Hornikx et al., 2012 [81]	Post hoc analysis of RCT; COPD patients in PR (n = 50)	Vitamin D3 100,000 IU monthly + PR, 3 months	Placebo + PR	Exercise capacity, muscle strength	↑ inspiratory muscle strength; ↑ VO_2_max	Small sample; short duration
Bjerk et al., 2013 [82]	Pilot RCT, severe COPD (n = 36)	Cholecalciferol 2000 IU/day, 6 wks	Placebo	SPPB, QoL, serum 25(OH)D	↑ 25(OH)D; no improvement in physical performance or QoL	Short duration; small sample; male-only cohort
Gouzi et al., 2019 [83]	RCT, COPD in PR (n = 64)	Nutritional supplementation [antioxidants]	Placebo + PR	Muscle endurance, strength, oxidative stress	↑ Muscle strength, ↓ muscle weakness; no added effect on endurance	Short duration; primary outcome negative
Weekes et al., 2009 [84]	RCT, stable COPD outpatients at nutritional risk (n = 59)	Dietary counselling + food fortification, 6 mo	Dietary advice leaflet	Nutritional status, dyspnoea, ADL, QoL, muscle strength	↑ Energy & protein intake, ↑ weight, ↑ QoL, ↓ dyspnoea; no change in muscle strength	Unblinded; small sample; no functional muscle gain

ADL: activities of daily living; COPD: chronic obstructive pulmonary disease; FFM: fat-free mass; IU: international units; ONS: oral nutritional supplements; PR: pulmonary rehabilitation; QoL: quality of life; RCT: randomized controlled trial; SPPB: Short Physical Performance Battery; VO_2_max: maximal oxygen uptake; wk: week; wks: weeks; mo: months; 25(OH)D: 25-hydroxyvitamin D; 6MWD: 6-min walk distance; ↑: increase; ↓: decrease. Arrows indicate direction of change compared with the control or comparator group.

**Table 4:** Pharmacologic and Adjunctive Non-Pharmacologic Interventions for Sarcopenia and Frailty in COPD.

Intervention Type	Author, Year	Population/Study Design	Intervention (Duration/Dose)	Comparator	Outcomes	Key Findings	Limitations
Anabolic Hormones	Casaburi et al., 2004 [98]	COPD men; RCT	Testosterone enanthate 100 mg IM weekly + PR, 10 wks	PR + placebo	LBM, leg strength, 6MWD	↑ LBM (~2 kg), ↑ strength	Adverse effects; men only
	Svartberg et al., 2004 [112]	COPD men w/ low testosterone; RCT	Oral testosterone undecanoate, 6 mo	Placebo	Strength, QoL	↑ handgrip strength	Small sample
	Burdet et al., 1997 [24]	COPD, malnourished; RCT	GH 0.05 mg/kg/day, 3 wks	Placebo	FFM, exercise tolerance	↑ FFM; no functional gain	Edema, glucose intolerance
	Dalton et al., 2011 [113]	Older adults; Phase II RCT	SARM (enobosarm) 3 mg/day, 12 wks	Placebo	LBM, stair climb power	↑ LBM & power	Limited COPD-specific data
Anti-inflammatory/Metabolic	Frost et al., 2007 [114]	COPD; Observational	Statins chronic use	Non-users	Muscle function, exacerbation	Mixed; possible ↓ inflammation	Myopathy risk
	Aaron et al., 2013 [115]	RCT, acute COPD exacerbation	Etanercept 50 mg SC (baseline + 1 wk)	Prednisone 40 mg/day ×10 d	FEV_1_, treatment failure, dyspnoea, QoL	No superiority over prednisone; similar FEV_1_, failure rates, QoL	Small sample; no long-term outcomes; infection risk
Myostatin/Emerging Molecules	Polkey et al., 2019 [116]	Phase II RCT, COPD with low muscle mass	Bimagrumab 30 mg/kg IV, 24 wks	Placebo	Thigh muscle volume, 6MWD, strength	↑ muscle volume (~5–8%); no significant ↑ 6MWD	Limited functional benefit; short duration; cost
	Coats et al., 2016 [117]	Chronic illness (COPD); Phase II	Espindolol	Placebo	Weight, FFM, strength	↑ FFM & strength	Limited COPD-specific evidence; safety unknown
	Gilson et al., 2009 [118]	Preclinical COPD models	Follistatin gene therapy	Control	Muscle hypertrophy	↑ muscle size	Preclinical only
Respiratory-Targeted	O’Donnell et al., 2004 [119]	COPD; RCT	LABA (salmeterol) 12 wks	Placebo	Exercise tolerance, dyspnea	↓ hyperinflation, ↑ tolerance	Minimal direct anabolic effect
	Brusasco et al., 2003 [120]	COPD; RCT	LAMA (tiotropium) 6 mo	Placebo	6MWD, QoL	↑ distance, QoL	Muscle outcomes indirect
	Fishman et al., 2003 [108]	Severe emphysema; RCT	LVRS vs medical therapy	Medical therapy	QoL, exercise, survival	↑ QoL, exercise; survival benefit	High morbidity; strict criteria
	Clini et al., 2002 [121]	COPD hypercapnia; RCT	Nocturnal NIV	Usual care	QoL, exercise	↑ rehab tolerance, QoL	Adherence issues
NMES	Vivodtzev et al., 2006 [70]	Severe COPD unable to exercise; RCT	NMES 35–50 Hz, 30 min/day, 6 wks	Sham NMES	Quadriceps strength, endurance	↑ strength, mobility, QoL	Small sample; equipment dependent
	Sillen et al., 2008 [71]	COPD in rehab; RCT	NMES + PR, 8 wks	PR alone	Muscle mass, 6MWD	↑ strength & walking	Response variability
Psychosocial/Cognitive Support	de Godoy & de Godoy, 2003 [122]	COPD w/ depression; RCT	CBT 12 wks	Usual care	Depression, QoL	↓ depression, ↑ QoL	Small sample
	Güell et al., 2006 [123]	COPD in PR; RCT	Psychosocial support + PR	PR alone	Adherence, exercise	↑ adherence, ↓ anxiety	Limited generalizability
Fall Prevention/Functional Independence	Beauchamp et al., 2013 [124]	COPD, balance impaired; RCT	Balance + strength 12 wks	Usual PR	Falls, balance, 6MWD	↓ falls, ↑ balance & function	Short follow-up
	Mak et al., 2022 [125]	Older adults; RCT	Single-task, dual-task, and analogy training; 12 sessions	Standard training	Gait, balance, mobility, fear of falling	↑ Gait & balance in all groups; AG superior for single- & dual-task walking	Short-term follow-up;
	Lopez-Lopez et al., 2020 [126]	RCT, hospitalized severe COPD	Daily individualized self-management + physiotherapy + NMES	Usual care	HRQoL, functionality	↑ HRQoL & function; best outcomes in self-management group; maintained at 3 months	Small sample; short follow-up

AG: analogy group; CBT: cognitive behavioral therapy; COPD: chronic obstructive pulmonary disease; FFM: fat-free mass; GH: growth hormone; HRQoL: health-related quality of life; LABA: long-acting β_2_-agonist; LAMA: long-acting muscarinic antagonist; LBM: lean body mass; NMES: neuromuscular electrical stimulation; NIV: non-invasive ventilation; PR: pulmonary rehabilitation; QoL: quality of life; RCT: randomized controlled trial; SARM: selective androgen receptor modulator; 6MWD: 6-min walk distance; ↑: increase; ↓: decrease.

## Data Availability

No new data were created or analyzed in this study. Data sharing is not applicable to this article.

## Abbreviations

ActRIIB, Activin Type II Receptor; AMPK, AMP-activated protein kinase; Bi-PAP, bilevel positive airway pressure; BLVR, bronchoscopic lung volume reduction; CGA, comprehensive geriatric assessment; COPD, chronic obstructive pulmonary disease; CPAP, continuous positive airway pressure; DHA, docosahexaenoic acid; EAA, essential amino acids; EPA, eicosapentaenoic acid; GH, growth hormone; HMB, β-hydroxy β-methylbutyrate; ICS, inhaled corticosteroid; LABA, long-acting β_2_-agonist; LAMA, long-acting muscarinic antagonist; LTOT, long-term oxygen therapy; LVRS, lung volume reduction surgery; MPS, muscle protein synthesis; NETT, National Emphysema Treatment Trial; NIV, non-invasive ventilation; NMES, neuromuscular electrical stimulation; ONS, oral nutritional supplements; SPPB, short physical performance battery; PR, pulmonary rehabilitation; PUFAs, polyunsaturated fatty acids; SARMs, selective androgen receptor modulators; 6MWT, six-minute walk test; TNF-α, tumor necrosis factor-α.
